# Supplementary material for: Colour change and behavioural choice facilitate chameleon prawn camouflage against different seaweed backgrounds
Source: Commun Biol. 2019 Jun 21;2:230. doi: 10.1038/s42003-019-0465-8 (PMC6588621; doi:10.1038/s42003-019-0465-8)
Supplement: Supplementary file 1 — Description of Additional Supplementary Files [file 42003_2019_465_MOESM1_ESM.pdf]

Description of Additional Supplementary Materials
File File Name: Supplementary Data 1

Description:

Initial Camouflage

Results of visual discrimination models (colour JNDs) to both goby and pollack vision. Showing green and red chameleon prawn body coloration compared to that of samples of the seaweeds green sea lettuce and red dulse.

Colour Change

Modelled cone catch response data corresponding to green and red chameleon prawns for both goby (long-wave sensitive [lws], medium-wave sensitive [mws] and short-wave sensitive [sws] cones) and pollack (long-wave sensitive [lws] and short-wave sensitive [sws] cones) over the 30 day experimental period. Hue values were calculated from relative proportions of cone catch data for each visual system (goby - sw/(lw+mw), pollack - sw/lw). Additionally, showing results of visual discrimination models (colour JNDs) to both fish visual systems over the 30 day period. Showing green prawn body coloration compared to that of samples of red dulse and red prawn body coloration compared to that of samples of green sea lettuce.

Behavioural Choice

Results of 10 minute behavioural choice trails where green and red chameleon prawns were given a choice between red dulse and green sea lettuce seaweed substrates in a y-choice decision chamber.
